# Introduction to the special issue: evolutionary and biopsychosocial perspectives on sickness communication

**DOI:** 10.1093/emph/eoae005

**Published:** 2024-03-25

**Authors:** Eric C Shattuck, Chloe C Boyle

**Affiliations:** Department of Anthropology, Florida State University, Tallahassee, FL, USA; Department of Psychiatry and Biobehavioral Sciences, Norman Cousins Center for Psychoneuroimmunology, Semel Institute for Neuroscience and Human Behavior, David Geffen School of Medicine, University of California, Los Angeles, CA, USA

**Keywords:** sickness, signaling, behavioral immune system, disgust, evolution of healthcare

## Abstract

Here, we introduce the EMPH special issue on Evolutionary and Biopsychosocial Perspectives on Sickness Communication. This Commentary provides an overview of each article and places them in the wider context of sickness as a social phenomenon with verbal and nonverbal signals. This Commentary, and the special issue, in general, calls for greater attention to these signals that can affect pathogen transmission and may be at the evolutionary root of our caregiving systems and behaviours.

This Special Issue of *EMPH* emerged from a symposium organized for the 2021 annual meeting of the International Society for Evolution, Medicine and Public Health. The COVID-19 pandemic highlighted—and continues to do so—the importance of reliable and accurate signals of infectious disease. These signals include early self-assessed sensations of sickness, which can lead to self-treatment and small changes in behaviour to minimize contact with others. They also encompass overt internal and external signs and symptoms that are obvious to others and diagnosable.

These sensations, signs and symptoms are our first indications of sickness and, in an ideal world, ought to motivate us to isolate, rest and seek treatment. Evolved behavioural manifestations of sickness (i.e. sickness behaviour), driven by inflammation, encourage us to do exactly this. And growing evidence supports the existence of a behavioural immune system, a suite of psychological capabilities for recognizing and responding to disease cues in the environment and in others. Intriguingly, activation of the behavioural immune system seems to also activate the biological immune system[1]. Put simply, human sickness is a social phenomenon awash with internal and external signals, each of which can elicit changes in behaviour and immune activity in both the sender and receiver. The existence of these sickness signals is relevant to pathogens too; pathogens that can avoid or delay activating these signals benefit from increased transmission (as in the case of SARS-CoV-2) [2]. As such, the study of these signals—their nature and mechanisms, their effects on signal recipients, and the broader social contexts that mould their transmission—is a key element of public health, albeit an underdeveloped one.

The papers collected here represent a broad approach to these questions using a variety of methods, including psychological experiments and induction of inflammatory and sickness states, national and international surveys and agent-based modelling. In addition to focussing on the nature of the signals themselves, several papers consider the implications of these signals and the activation of their underlying psychological architecture on social outcomes, as well as the role of sickness signals in the evolution of human medicine.

Focusing first on mechanisms, Paola Bressan uses publicly available data from India and the USA to demonstrate that our first impressions of strangers are informed by the behavioural immune system. Bressan’s results indicate that perceived positive qualities (intelligence, for instance) increased with estimated facial health and similarity to individuals in the local community. While negative qualities (unfriendliness, laziness and so on) similarly decreased with estimated facial health, they also increased as a function of the perceiver’s pathogen disgust sensitivity and self-reported illness at the time of rating the images. Moreover, the effect of estimated facial health on decreased negative attributions was three times higher in the USA than in India, where the latter country is taken to represent a greater risk of infection in general. In other words, in those countries where infection risk is relatively high (e.g. India), health cues are less able to mitigate the negative response to strangers. Taken together, these results highlight the importance of local pathogen contexts in moderating the strength of behavioural immune responses.

Another key moderator is sex differences. Tognetti *et al.* tested variation in sickness signal detection based on sex and disease-related traits in a large, online study (*n* = 683). The authors hypothesized that women may be more sensitive to sickness cues given their greater investment in offspring during human evolutionary history. Notably, this study uses pairs of composite photographs taken during a control condition and an experimental sickness condition, rather than manipulating faces by changing skin colour or adding an explicit disease cue, such as a rash. In exploratory analyses, women indeed were more accurate—and faster—than men in distinguishing between sick and healthy faces. Higher disgust sensitivity to body odour, but not perceived vulnerability to disease, health anxiety or self-reported health, was also associated with a greater likelihood of classifying faces as sick, indicating a bias towards perceiving sickness cues.

Stephanie Anja Juran and Arnaud Tognetti provide two papers that help expand our understanding of the behavioural immune system into not only sight but also odour, and consider additional sources of variation in behavioural immune responses. First, Tognetti *et al.* find that humans can discriminate between body odours from healthy and sick individuals during natural, rather than experimental, infection. In this, humans are like many other animals from diverse genera that can distinguish the sick from the well based on scent. All five senses, therefore, appear to be engaged in detecting sick individuals: in addition to the present work on scent, Bressan and many other authors have shown that changes in skin coloration and other visible signs of illness (e.g. changes in gait) lead to avoidance and/or negative assessments; touch can be used to assess fever; experimentally induced inflammation is associated with changing breathing patterns in men; and taste has been historically used to distinguish between healthy and diabetic urine, though thankfully diagnostic techniques have improved in their modality and precision. The extent to which makeup, deodorant and similar hygienic products can mask these signals is an open question. The redundancy of signals across the senses should work in our favour here, as changes in scent and skin coloration could be covered up, but changes in breathing patterns or gait are much less likely to be hidden. Moreover, as shown by Tognetti *et al.*, detection by odour and facial cues is only slightly better than chance. Redundant or overlapping signals could increase the likelihood of perceiving sickness or reduce the likelihood of false positives. Future research here should consider a holistic approach to these signals rather than studying them in isolation.

Juran *et al.* find that exposure to ‘disgusting’ odours was associated with increases in levels of one of two immune markers (salivary tumour necrosis factor-alpha [TNF-a], but not secretory immunoglobulin A [sIgA]). Results such as these lend support to the argument that the behavioural immune system is important in maintaining health; in addition to driving avoidance, it appears to be able to launch an anticipatory increase in immune function. While the stimuli used in this study were not direct indicators of sickness but rather linked with potential pathogen exposure (e.g. skatole, a faecal odour), an important next step will be to link human sickness odours (see above) with such anticipatory immune responses and to identify the extent to which such anticipatory responses have direct relevance for health or align with blood immune markers.

These papers on visual and scent cues of sickness represent some initial steps towards a fuller understanding of sickness signals—what form they take in humans, their relationship to immunity (if any) and importantly, sources of variation in their transmission and reception. While the behavioural immune system may be an evolved adaptation to the challenge of pathogen transmission in a socially living species, we should not expect it to act the same everywhere. Like any biological system operating in a socially complex, biocultural species, we must expect that its operation will be shaped by higher-level social contexts.

Mícheál de Barra, Kawthar Hakimy and Marijn de Bruin explore the social context of ambiguous sickness signals above and beyond communicating (consciously or unconsciously) sickness to others. In addition to avoidance, which is where much of the research on the behavioural immune system focuses, sickness signals can elicit caregiving from close others or professional strangers. Sickness, as a physiological state, has been theorized to carry with it certain affordances [3]. These are primarily a temporary release from normal social roles, with the expectation that the sick individual undergoes treatment. As with any signal with appreciable benefits (secondary sexual characteristics/biological quality in the context of sexual selection, for instance), there is a chance that some individuals will fake or exaggerate their sickness signals. de Barra and colleagues conducted three studies that consider honest signals of sickness. Using a series of vignettes, they find that participants are more likely to provide care to those people who undergo medical treatments (and particularly aversive treatments), particularly for illnesses that lack visible sickness cues (e.g. reports of back pain). Additionally, participants in a separate study indicated that they would be more willing to accept treatment (and again, especially aversive treatments) if they felt others were questioning the legitimacy of their illness. These results highlight the sometimes complex relationship between signaller and receivers and that medical treatments can act as additional, extracorporeal signals of sickness. As the authors note, the incentives inherent in this system—that (aversive) treatments lead to less stigma and more legitimization of sickness—may lead people to consume more or riskier medical treatments than is strictly necessary. This has particular salience in the USA, where sick leave is not universal and there persists a culture of presenteeism, or continuing to attend work/school while sick [4].

Amanda C de C Williams similarly reviews signals of chronic and acute pain across human and animal social contexts. Pain has multiple functions, ranging from immediate reflex withdrawal to longer-term behaviours that promote healing through conservation of energy and heightened threat vigilance. In dangerous environments, such shifts in behaviour provide clear survival advantages. However, the associated risks of demotivation and immobility represent a clear trade-off in terms of maintaining activities required for survival. The ‘mismatch’ hypothesis of chronic pain is described, whereby, when survival needs can be met by conspecific help, such immobility and heightened vigilance can serve to prolong pain. Yet, as with illnesses, chronic pain sufferers often report being stigmatized and viewed with suspicion. Indeed, results from the de Barra study indicate that individuals with chronic pain report being more likely to undergo aversive treatments to legitimize their pain to others. More generally, Williams calls for better attention to be paid to signals of pain as well as the social contexts in which those signals are transmitted and received. This emphasis on recognizing and responding appropriately to pain signals is a central theme of this special issue, underscoring the importance of integrating knowledge of pain’s social dimensions into interpersonal dynamics, healthcare practices and public health strategies.

Expanding our perspective beyond individuals and dyads, van Diepenbeek and Kessler consider the role of authoritarian and collectivistic attitudes and the behavioural immune system in support of COVID-19 lockdowns in the UK. Following the smoke-detector principle, wherein false positives are less costly than false negatives, the behavioural immune system is likely to be oversensitive to sickness cues and signals, although this could vary as a function of social values. Individual variation in fear of contagion could drive shifts in attitudes during epidemics and pandemics, namely toward authoritarian regulations as a means of protecting individual and public health or a reliance on community resilience in the face of these challenges rather than top-down impositions. Contrary to their hypotheses, however, the authors found that COVID-19 worry was related to supporting lockdown restrictions and enforcement directly, independent of authoritarian or collectivistic values.

Finally, Sharon Kessler, Robert Aunger and Bethany Gilbert provide us with two articles that together consider the evolution of human caregiving and its implications for the immune system, current healthcare systems and social behaviours more broadly. Of course, without the types of signals described above, caregiving is unlikely to have evolved and developed. First, Gilbert and Kessler use agent-based modelling to test the hypothesis that advancements in human caregiving over evolutionary time may have actually modified our immune responses to shift towards acquired relative to innate immune responses. Data generated from computer simulations of a hominin population were consistent with the hypothesis that a higher rate of caregiving was associated with a higher prevalence of acquired immunity. Moreover, this increased caregiving, despite higher social contact among conspecifics, was associated with an overall decrease in disease prevalence. The authors note that these conditions likely further support the emergence of cooperative care despite its potential costs to the individual.

Kessler and Aunger divide human healthcare behaviours between behaviours directed at caring for individuals (self, kin or strangers) and those at protecting the wider community and trace their possible evolutionary roots. From these, we derive modern medicine and public health practices and systems. The COVID-19 pandemic highlights some aspects of their proposed framework, such as the reliance on the evolutionarily older kin care when stranger care (i.e. medical professionals) systems become overwhelmed or concerns about infection are particularly high, or the inherent evolutionary conflict between governmental measures focussed on population health (e.g. lockdowns) and their costs to individual and kin fitness, among others. This conflict between the health of heterogenous human groups and individuals that drives attention towards local communities at the expense of others unlike us is a significant vulnerability for global health in a time where pathogens travel the globe within hours and days.

It is our hope that this special issue makes a significant contribution to our understanding of human sickness as a social phenomenon. We are continually sending and receiving accurate and reliable signals of sickness and health, which are at the heart of caregiving as a behaviour and our medical systems. However, how we choose to act—or not—on these signals is likely dependent on multiple factors, including individual histories and personality as well as social contexts. There are times when concealing sickness is advantageous for individuals (such as when we wish to continue working despite illness) and times when exaggerating sickness may be better, as when we use sickness as an excuse to avoid work or other social interactions [5]. It is decisions like these, conscious or unconscious, that help shape disease transmission networks and could help determine the scope of epidemics and pandemics.
